# HBx-upregulated lncRNA UCA1 promotes cell growth and tumorigenesis by recruiting EZH2 and repressing p27Kip1/CDK2 signaling

**DOI:** 10.1038/srep23521

**Published:** 2016-03-24

**Authors:** Jiao-Jiao Hu, Wei Song, Shao-Dan Zhang, Xiao-Hui Shen, Xue-Mei Qiu, Hua-Zhang Wu, Pi-Hai Gong, Sen Lu, Zhu-Jiang Zhao, Ming-Liang He, Hong Fan

**Affiliations:** 1 Department of Medical Genetics and Developmental Biology, Medical School of Southeast University, The Key Laboratory of Developmental Genes and Human Diseases, Ministry of Education, Southeast University, Nanjing, China; 2 The First Affiliated Hospital with Nanjing Medical University, Nanjing, China; 3 CUHK Shenzhen Research Institute, Shenzhen, China.; 4 Department of Biomedical Sciences, City University of Hong Kong, Hong Kong

## Abstract

It is well accepted that HBx plays the major role in hepatocarcinogenesis associated with hepatitis B virus (HBV) infections. However, little was known about its role in regulating long noncoding RNAs (lncRNAs), a large group of transcripts regulating a variety of biological processes including carcinogenesis in mammalian cells. Here we report that HBx upregulates UCA1 genes and downregulates p27 genes in hepatic LO2 cells. Further studies show that the upregulated UCA1 promotes cell growth by facilitating G1/S transition through CDK2 in both hepatic and hepatoma cells. Knock down of UCA1 in HBx-expressing hepatic and hepatoma cells resulted in markedly increased apoptotic cells by elevating the cleaved caspase-3 and caspase-8. More importantly, UCA1 is found to be physically associated with enhancer of zeste homolog 2 (EZH2), which suppresses p27Kip1 through histone methylation (H3K27me3) on p27Kip1 promoter. We also show that knockdown of UCA1 in hepatoma cells inhibits tumorigenesis in nude mice. In a clinic study, UCA1 is found to be frequently up-regulated in HBx positive group tissues in comparison with the HBx negative group, and exhibits an inverse correlation between UCA1 and p27Kip1 levels. Our findings demonstrate an important mechanism of hepatocarcinogenesis through the signaling of HBx-UCA1/EZH2-p27Kip1 axis, and a potential target of HCC.

Hepatocellular carcinoma (HCC), the fifth most common cancer worldwide, is the second leading cause of cancer death in men^1^. Chronic infection by hepatitis B virus (HBV) is a major risk factor for hepatocarcinogenesis^2^,^3^. The X protein (HBx) encoded by HBV is believed to be the main protein in the process of HBV-induced oncogenesis^4^,^5^. It has been well documented that HBx induces tumours in transgenic animals^6^,^7^. However, the precise mechanisms underlying HBx-mediated hepatocarcinogenesis remain an unsolved mystery.

Viral oncoprotein-induced epigenetic alterations, including dysregulation of DNA methylation, histone modifications and non-coding RNAs, have been reported to be critical for induction of carcinogenesis and tumorigenesis^8^,^9^,^10^. Accumulated evidence has indicated that HBx affects DNA methylation through selectively promoting regional hypermethylation of specific tumour suppressor genes (TSG) by upregulating of DNMT1, DNMT3A1, and DNMT3A2^11^. HBx also induces hypomethylation of distal intragenic CpG islands by recruiting HDAC1 to the promoter of DNMT3L and DNMT3A to downregulate their expressions^12^. The status of histone modifications is associated with gene transcriptional activity, such as histone methylations or acetylations. HBx recruited CREB-binding protein (CBP)/p300 and HDAC1 to the promoters for inducing or suppressing target gene expression^13^,^14^,^15^. It has been shown that HBx is capable of upregulating methyltransferase SMYD3 and its trans-activated oncogenes^16^. Furthermore, HBx has also been found to involve in the alteration of miRNA expression profile^11^,^17^,^18^.

Long noncoding RNAs (lncRNAs), pervasively transcribed in the genomic loci but exhibited tissue and cell type specific expression patterns, is a broad definition that encompasses different classes of RNAs that transcripts longer than 200 nucleotides without evident protein coding potential^19^,^20^. They play critical roles in diverse biological processes, including cell proliferation, apoptosis, invasion, metastasis and angiogenesis^21^,^22^,^23^. Compared with miRNAs, lncRNAs display much more complicated regulatory effects on gene expression through epigenetic silencing, mRNA splicing, lncRNA–miRNA interaction, lncRNA–protein interaction and lncRNA–mRNA interaction^24^,^25^,^26^,^27^,^28^,^29^. However, the effects of HBx on lncRNAs expression and the underlying molecular mechanisms in hepatocarcinogenesis have not been understood.

In the present study, we determined the lncRNA profiles in HBx-expressing and non-expressing cells by lncRNA microarrays. UCA1 was significantly upregulated by HBx, and its effects and underling mechanisms in HCC were further investigated.

## Results

### The lncRNAs profiles upon HBx expression in hepatocytes

**Figure 1:**
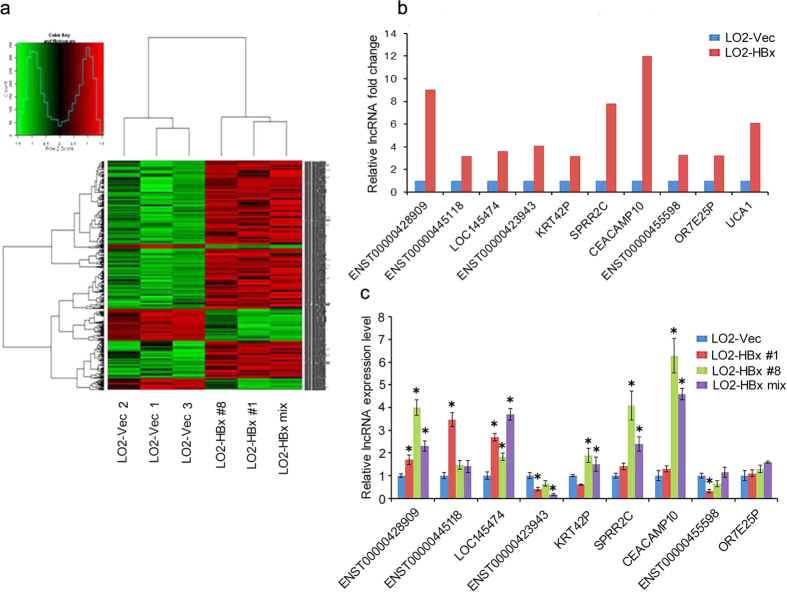
Differential expression profile of lncRNAs in HBx transfected LO2 cells. (**a**) Hierarchical clustering analysis of lncRNAs. 379 up-regulated lncRNAs (fold change ≥2, *P* < 0.05) and 97 down-regulated lncRNAs (fold change ≥4, *P* < 0.05) were differentially expressed in HBx-expressing cells. The upregulated lncRNAs are represented in red, and the downregulated lncRNAs are displayed in green. The experiments were conducted in three biological replicates. (**b**) The fold change of lncRNA in microarray. (**c**) Nine differentially expressed lncRNAs randomly selected from panel (**a**) were validated by real-time PCR. Two LO2-HBx stable cell lines (LO2-HBx #1 and LO2-HBx #8) and LO2-HBx mixed stable cells were used to validate lncRNA profiles. Statistically significant differences are reported (Student t-test) for three independent experiments. ^*^P < 0.05.

To examine whether lncRNAs are regulated by HBx, we employed microarray analysis to reveal lncRNA profiles in LO2-HBx and control LO2-Vector cells. Results showed that there were 379 up-regulated and 724 down-regulated lncRNAs in LO2-HBx cells with at least 2-fold change (Fig. 1a). To further verify the microarray data, 10 lncRNAs with raw intensity >300 and >3-fold change were randomly selected and validated by real-time PCR. We showed that the expression tendency of most lncRNAs (7/10) was consistent with the microarray data (Fig. 1b,c).

### Upregulation of lncRNA UCA1 by HBx in hepatocytes

**Figure 2:**
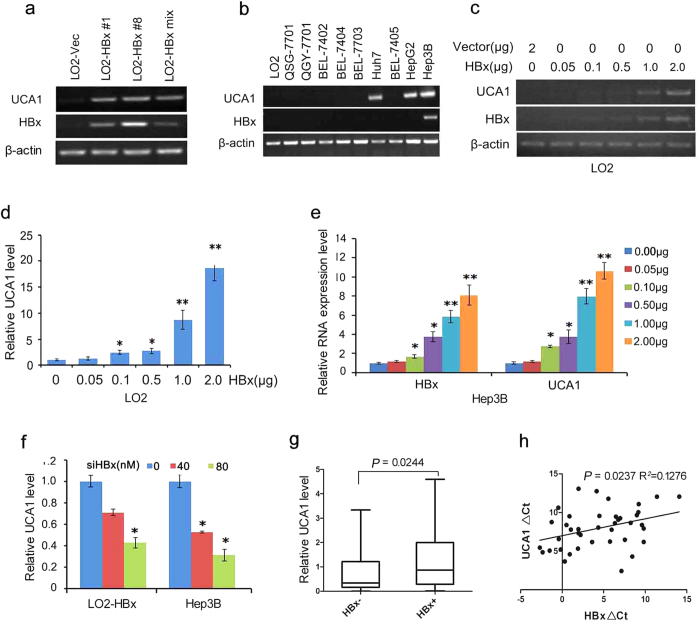
Induction of lncRNAs UCA1 expression by HBx. (**a**) The levels of HBx mRNA and UCA1 in LO2-HBx stable cells measured by conventional RT-PCR. (**b**) The levels of HBx mRNA and UCA1 detected in 10 liver cell lines by RT-PCR. (**c**,**d**) UCA1 levels in LO2 cells transiently transfected with an HBx-expressing plasmid pcDNA4.0-HBx at different doses. UCA1 was examined either by conventional RT-PCR (**c**) or real-time PCR (**d**). (**e**) The relative levels of HBx mRNA and UCA1 in Hep3B cells after transfected with different dose of an HBx-expressing plasmid. The value of control was set as 1. (**f**) The relative levels of HBx mRNA and UCA1 in LO2-HBx and Hep3B cells after transfected transiently with HBx-siRNA or negative control siRNA. β-actin was used as internal control in above experiments. Statistically significant differences are reported (Student t-test) for three independent experiments. ^*^P < 0.05; ^**^P < 0.01. (**g**) UCA1 levels measured by real-time PCR in 60 paired tumour and adjacent nontumor tissues. The HCC specimens are divided into two sub-groups based on their relative HBx mRNA status (HBx negative and positive group). Horizontal lines in the box plots represent the median; the boxes represent the interquartile range, and whiskers represent the 2.5th and 97.5th percentiles. Wilcoxon’s signed-rank test was used in this study, *P* = 0.0244. (**h**) The inverse correlation of levels of HBx mRNA and UCA1 in HBx positive samples. The ΔCt values (normalized to β-actin) were subjected to Pearson’s correlation analysis (R^2^ = 0.1276, *P* = 0.0237).

LncRNA UCA1 was selected for further study because its expression level was almost undetectable by RT-PCR in LO2 cells, while it was dramatically elevated in HBx-expressing cells (Fig. 2a). UCA1 levels seemed to be positively correlated with HBx expression levels in the tested HBx-expressing cells (Fig. 2a and Fig. S1a). We have transiently upregulated HBx expression in HBx-lacking cell lines QSG-7701 and detected the expression levels of HBx and UCA1. And the results showed that HBx upregulated UCA1 expression (Fig. S2a). To further quantify the expression difference of UCA1 in HBx negative and positive cells, we conducted real-time PCR experiments. As shown in supplementary Fig. S1b, UCA1 expression level was much higher in HBx-LO2 stable cells than that in LO2-vector cells. UCA1 level was elevated by HBx at least 8- to 14-fold in the tested cells. We then examined the mRNA levels of HBx and UCA1 in 9 other hepatoma cell lines by RT-PCR. We showed that UCA1 expressed in a group of hepatoma cells, i.e., Huh7, HepG2 and Hep3B cells. Among them, the mRNA of HBx was only detected in Hep3B cells; however, Huh7 and HepG2 also have UCA1 expression but without HBx expression (Fig. 2b). To further investigate if the dose of HBx would affect UCA1 expression, LO2 (UCA1 negative) and Hep3B (UCA1 positive) cells were transiently transfected with the pCDNA4.0-HBx and pCDNA4.0 vectors. We found that UCA1 was upregulated by HBx in a dose-dependent manner (Fig. 2c–e). As expected, UCA1 level was significantly reduced by HBx knockdown in LO2-HBx and Hep3B cells (Fig. 2f).

### Correlation of UCA1 and HBx levels in HCC specimens

To investigate whether UCA1 expression is correlated with HBx levels in HCC tissues, we examined the mRNA levels of HBx and UCA1 in 60 paired tumour and non-tumour specimens by real-time PCR. We evaluated the correlation between UCA1 and the clinicopathological characteristics. Although no significant correlation was observed among UCA1 to patients’ age, gender, tumour size, AFP level, and the differentiation degree of tumours; strikingly, a significant association was observed between UCA1 and HBx (*P* = 0.028) (Table 1). More importantly, UCA1 expression was significantly higher in HBx positive group as compared with HBx negative group (*P* < 0.001, Fig. 2g). The level of UCA1 was positively correlated with the mRNA level of HBx in the HCC tissues (R^2^ = 0.1276, *P* = 0.0237, Pearson’s correlation, Fig. 2h).

### Promotion of cell growth by ectopic expression of UCA1 in HCC cells

**Figure 3:**
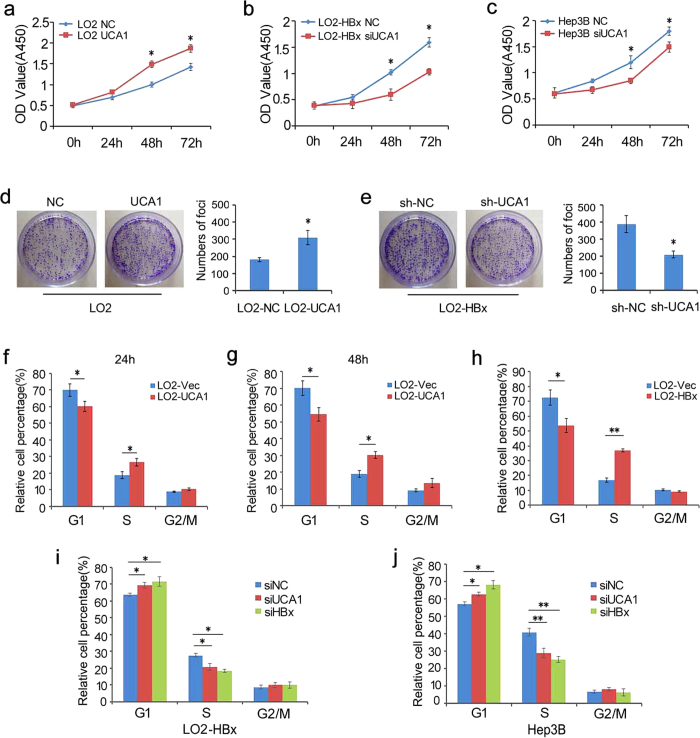
The Role of UCA1 in cells proliferation and cell cycle *in vitro*. Cell proliferation was measured by Cell Counting Kit-8 (CCK-8) assays (a to c). (**a**) LO2 cells were transfected with plasmids pcDNA3.1-UCA1 (**a**); (**b,c**) LO2-HBx cells (**b**) or Hep3B (**c**) cells were transfected with UCA1-specific siRNA. (**d**,**e**) colony formation assays. Representative images of colony formation of LO2 cells transfected with plasmids pcDNA3.1-UCA1 (**d**) and LO2-HBx stably transfected with pSUPER-EGFP-shUCA1 (**e**) are shown. Colonies were stained with crystal violate and counted after fourteen days. The colonies were counted and are depicted in a bar chart (right panel). All above the values indicate the mean ± s.d. for three separate experiments (^*^P < 0.05, independent Student t-test). (**f**–**h**) Cell cycle distribution in LO2 cells. Cell cycle distribution was examined by fluorescent activated cell sorting (FACS) assays in LO2 cells transiently transfected with either a UCA1-expressing plasmid (**f,g**) or an HBx-expressing plasmid (**h**) after stained with propidiumiodide. The bar chart represents the relative percentages of cells in each phase. The values indicate the mean ±s.d. for three separate experiments (^*^P < 0.05, ^**^P < 0.01, Student t-test). (**i,j**) Cell cycle distribution in LO2-HBx stable cells and Hep3B cells upon UCA1 or HBx knocked down by specific siRNAs. From (**h–j**), Flow cytometry was 24h post-transfection. The bar chart represents the relative percentages of cells in each phase. The values indicate the mean ± s.d. for three separate experiments (^*^P < 0.05, ^**^P < 0.01, Student t-test).

The function of UCA1 in hepatocarcinogenesis remains to be elucidated. We first tested if UCA1 would affect the cell growth. As shown, UCA1 significantly promoted cell proliferation when it was transiently overexpressed in LO2 (Fig. 3a). And, cell proliferation ability was improved in QSG-7701-HBx with UCA1 upregulated (Fig. S2b). Conversely, the cells showed a markedly reduced proliferation rate when UCA1 was knocked down by specific siRNAs in LO2-HBx, Hep3B and HepG2 cells (Fig. 3b,c and Fig. S2c). To further confirm our observation, we performed colony-formation assays. The number of colonies was significantly increased when UCA1 was ectopically expressed in LO2 cells (Fig. 3d) while markedly decreased when UCA1 was knocked down in LO2-HBx cells (Fig. 3e). Furthermore, we showed that ectopic expression of UCA1 or HBx significantly increased the percentage of LO2 cells in the S phase (Fig. 3f–h); whereas knockdown of UCA1 or HBx resulted in a significant reduction in the percentage of cells in the S phases and increased cell percentages in the G1 phases in LO2-HBx and Hep3B cells (Fig. 3i,j) indicating a contribution of UCA1 in G1/S transition.

### Cell apoptosis induced by knockdown of UCA1 expression

**Figure 4:**
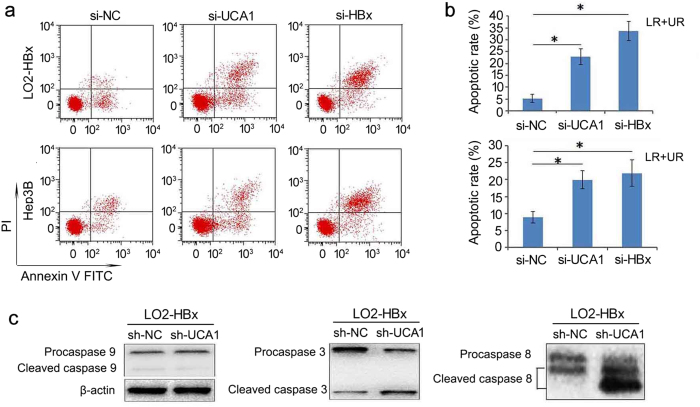
Apoptosis induced by knockdown of UCA1 in hepatic HCC cell lines. (**a**) Flow cytometry analysis of cell apoptosis in LO2-HBx and Hep3B cells transfected with si-NC, si-UCA1 and si-HBx. Apoptotic cells were stained with FITC-labeled Annexin V and analysed by FACS assays. Knockdown of UCA1 and HBx resulted in significant increase of apoptotic cells when compared with control cells. (**b**) The apoptosis rate was calculated and depicted in a bar chart. The values indicate the mean ± s.d. for three separate experiments (Student t-test, ^*^P < 0.05). LR, early apoptotic cells; UR, terminal apoptotic cells. (**c**) Western blot analysis for cleaved caspase-9, caspase-3 and caspase-8 in LO2-HBx stable cells transfected with pSUPER-EGFP-shUCA1 and control vector cells, respectively. β-actin was used as a loading control. The data are representative of three independent experiments.

We further checked if UCA1 contributed to apoptosis in HBx-expressing cells. We stained the cells with Annexin V and measured the apoptotic cells by flow cytometry assay. We showed that knockdown of UCA1 or HBx resulted in an obvious increase of apoptotic cells number. There were 20.95% and 22.97% cells undergoing apoptosis 48 hours post-transfection with UCA1 siRNA treatment; while only 8.6% and 5.28% of apoptotic cells were detected in the control LO2-HBx and Hep3B cells, respectively (Fig. 4a,b). We then examined the status of caspases involved in the process of apoptosis. UCA1 decreased significantly elevated the cleaved caspase-3 and caspase-8 but not caspase-9 in LO2-HBx-shUCA1 cells (Fig. 4c). The density of signals from western blot were shown in supplementary Fig. S3.

### Suppression of p27 expression by UCA1

**Figure 5:**
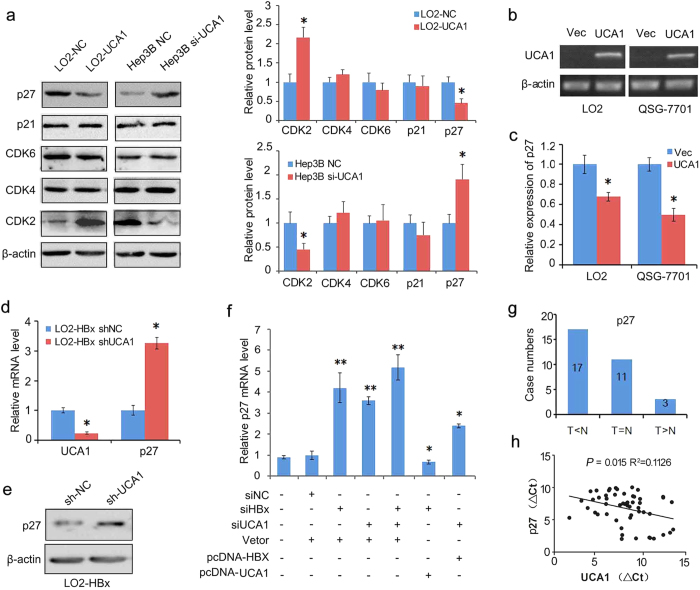
Suppression of p27 expression by UCA1 in HCC. (**a**) Protein levels of cyclin-dependent kinases (CDKs) and CDK inhibitors (CDKI) in LO2 cells with ectopically expressed UCA1 and in Hep3B cells with UCA1 knockdown. The relative protein levels were analysed by density assays and presented in a bar chart (right panel). (**b,c**) The UCA1 levels were examined by conventional RT-PCR (**b**) and p27 levels were examined by real-time PCR(c) in LO2 and QSG-7701 cells transiently transfected with UCA1 expressing or control vectors, respectively. (**d**) The levels of UCA1 and p27 mRNA were examined by qPCR in LO2-HBx cells stably transfected with pS-EGFP-shUCA1 and a control plasmid. (**e**) Knockdown of UCA1 expression upregulated p27 protein level was detected by western blot. (**f**) The levels of p27 mRNA were detected by real-time PCR in Hep3B cells transfected with indicated siRNA, HBx-, or UCA1-expressing plasmids, respectively. The ratio of mRNA copies of p27 to β-actin was set as 1 in the control groups in above experiments. Student t-test was conducted for statistical analysis. At least three independent experiments were conducted. ^*^P < 0.05; ^**^P < 0.01. (**g**) The relative p27 levels were assayed by real-time PCR in 31 HCC and pair-matched non-tumour tissues. (**h**) The Pearson’s correlation analysis showed a negative correlation of UCA1 and p27 mRNA expression (R^2^ = 0.1126, *P* = 0.015).

Cyclin-dependent kinases (CDKs) play key roles in G1 to S phase transition. Cell cycle dysregulation is often mediated by alterations of cyclin-dependent kinase (CDKs) activities. UCA1 was ectopically expressed in LO2 cells or endogenous UCA1 was silenced by a pool of small interfering RNAs in HBx-expressing Hep3B cells. We showed that ectopically expressed UCA1 upregulated CDK2 only without affecting CDK4 and CDK6 in LO2 cells. Consistently, knockdown of UCA1 in Hep3B cells decreased CDK2 only without elevating other CDKs (Fig. 5a), indicating that UCA1 specifically regulated CDK2 expression in hepatocytes. As CDK2 plays a crucial role in both cell cycle progression and apoptotic response^30^, and its activity is regulated by CDK inhibitors p21 and p27^31^. We hypothesized that UCA1 might enhance CDK2 expression by repressing p21 and p27 expression. Our results indeed showed that UCA1 suppressed p27 expression at both mRNA and protein levels, but did not affect p21 expression in both LO2 and Hep3B cells (Fig. 5a).

To reveal whether the suppression of p27 expression by UCA1 is a common phenomenon in hepatocytes, we transiently expressed UCA1 in LO2 and QSG-7701 cells (Fig. 5b). As expected, the expression of p27 was obviously down-regulated in these cells both in mRNA and protein level (Fig. 5c and Fig. S4). On the other hand, both the mRNA and protein levels of p27 were up-regulated by silencing the endogenous UCA1 with UCA1-specific shRNA (Fig. 5d,e).

It seemed that HBx displayed stronger effect for promoting G1/S transition (Fig. 3), whereas knockdown of HBx in HBx-expressing cells induced more cells undergoing apoptosis (Fig. 4). To further investigate the degree of suppressing p27 expression through UCA1, we carried out a comprehensive experiment. As shown in Fig. 5f, knockdown of UCA1 significantly increased the p27 expression in Hep3B cells but the degree of increase was a little bit less than that of knockdown of HBx. Simultaneous knockdown of HBx and UCA1 further promoted p27 expression. More interestingly, we found that silence of HBx with ectopic expressing of UCA1 reduced p27 to the base level while silencing UCA1 with ectopic expressing HBx only partly reduced p27 level as compared with either knockdown of UCA1 or HBx. Taking together, these data indicated that UCA1 was an important mediator of HBx-associated p27 expression.

### Inverse correlation between p27 and UCA1 in HCC patients

To evaluate the correlation between UCA1 and p27, we measured the mRNA levels of p27 and UCA1 in 31 paired tumour and adjacent nontumor tissues by real-time PCR. The expression of p27 was at lower level in 54.8% tumour tissues as compared to the non-tumour tissues (17/31) (Fig. 5g), and a significantly inverse correlation was observed between UCA1 and p27 levels (R^2^ = 0.1126, *P* = 0.015, Wilcoxon’s signed-rank test, Fig. 5h). These results revealed that UCA1 suppressed p27 expression in HCC.

### The physical association of UCA1 and EZH2 on p27 promoter

**Figure 6:**
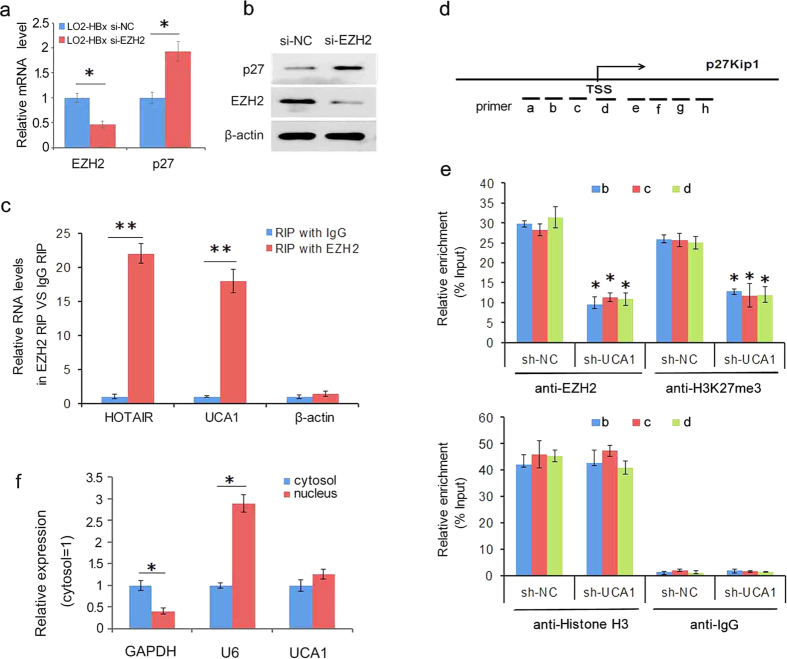
The physical association of UCA1 and EZH2 elevating p27 3MeK27H3 level in promoters. (**a**) The relative mRNA levels of EZH2 and p27 after transfection of EZH2-specific siRNA or scramble siRNA into LO2-HBx cells (si-NC or si-EZH2). The EZH2 and p27 mRNA levels were examined by real-time PCR (^*^P < 0.05, Student t-test). (**b**) Upregulation of p27 protein levels by knockdown of EZH2. (**c**) RNA-binding protein immunoprecipitation (RIP). The lysates of LO2-HBx cells were applied to co- immunoprecipitation with antibodies against EZH2 and IgG, then HOTAR, UCA1 and β-actin were detected by real-time PCR. The values indicate the mean ± SD. Statistically significant differences are reported for three independent experiments. ^**^P < 0.01. (**d,e**) ChIP assays. (**d**) primer sets conducted on p27 promoters. (**e**) Cell lysates from LO2-HBx cells without and with UCA1 knockdown were applied for immunoprecipitation using anti-EZH2, -H3K27me3, -Histone H3 and IgG antibodies. ChIP samples at the p27 promoter was quantified by real-time PCR. The ChIP results revealed that the EZH2 occupied across p27 promoters around the transcription start site (primers b, c and d) (upper), but without bind the region correspond to primer a, e, f, g and h (data not shown). Knockdown of UCA1 could not alter the H3 total levels in binding-regions (lower). The values indicate the mean ± SD. Statistically significant differences are reported for two independent experiments. ^*^P < 0.05. (**f**) UCA1 was detected both in the cytosolic and nuclear compartments (n = 2).

EZH2, the main subunit of polycomb repressive complex 2 component (PRC2), functions as a histone methyltransferase involved in silencing bunches of tumour suppressor genes by trimethylation of lysine 27 on histone H3 across promoters^32^. It was reported that EZH2 inhibits the expression of p27^33^,^34^. We postulated that the suppression of p27 expression by UCA1 might be mediated through recruiting EZH2 on p27 promoter. Knockdown of EZH2 in LO2-HBx cells increased p27 expression (Fig. 6a,b), indicating that EZH2 played an important role in HBx/UCA1-mediated p27 silence. We further performed RNA-binding protein immunoprecipitation (RIP) assay to explore whether UCA1 directly interacted with EZH2 using anti-EZH2 antibody in LO2-HBx cells. As compared to IgG control antibody, we observed a significantly higher enrichment of UCA1 and HOTAIR (a validated EZH2 binding lncRNA as positive control) with anti-EZH2 antibody, but no enrichment was found with negative control β-actin (negative control) (Fig. 6c). To show the specific-binding of UCA1 and EZH2, RIP assays were conducted in LO2-HBx cells with or without RNase treated (Fig. S5a). UCA1 was not enriched with anti-EZH2 antibody when treated with RNase. Results from ChIP analysis showed an effective immunoprecipitation from LO2-HBx cells because both EZH2 and H3K27me3 were depleted in the post-IP cell lysates (Fig. S5b). The ChIP results revealed that the EZH2 occupied across p27 promoters around the transcription start site (primers b, c and d) (Fig. 6d). Knockdown of UCA1 contributed to decrease the binding of EZH2 and H3K27me3 on p27 promoter without affect Histone H3 binding (Fig. 6e). The depletion of UCA1 was detected by qPCR in the nucleus of LO2-HBx cells (Fig. S5c). However, we did not observe apparent change of EZH2 and H3K27me3 binding on Foxc1 (a target of EZH2) promoter after knockdown of UCA1 expression (Fig. S5d). To confirm whether UCA1 dependent repression of p27 is mediated by EZH2, a comprehensive experiment was conducted. Results indicated that double siRNA treatment (UCA1/EZH2) showed the same p27 expression as compared to single siRNA treatments, and overexpression of UCA1 in the absence of EZH2 failed in P27 repression (Fig. S5e). Finally, we showed that UCA1 not only localized in the cytosol, but also existed in the nuclear compartments as well (Fig. 6f). These results demonstrated that, at least in part, UCA1 epigenetically silenced p27 expression by physical association with EZH2 in cancer cells.

### Suppression of tumorigenesis by knockdown of UCA1 in Hep3B cells

**Figure 7:**
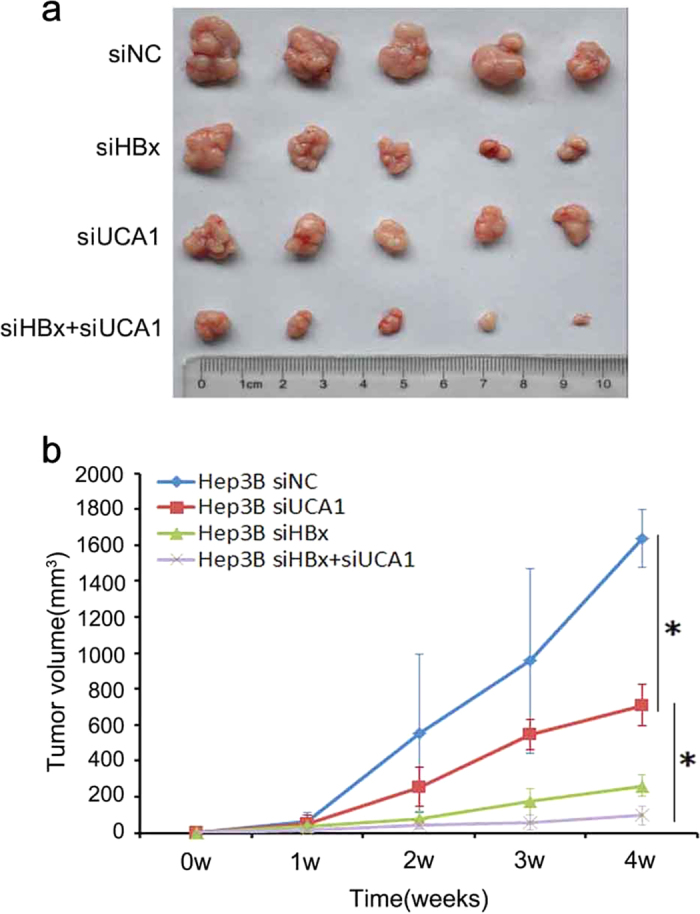
Inhibition of tumour growth by knockdown of UCA1 in a xenograft mouse model. (**a**) Actual tumour size after removal from mice which is injected with cells transfected by siUCA1, siHBx and control siRNA. (**b**) Tumour volumes were calculated after injection every week. Bars indicate S.D. The tumour growth curve shows that knockdown of UCA1 significantly inhibited tumour growth in the mice. Asterisk indicates a significant change (^*^P < 0.05). Data are the mean ± SD.

We postulated that UCA1 might also play crucial role in tumorigenesis in HCC development. We injected Hep3B cells without/with either knockdown of UCA1, HBx alone or simultaneous silence of UCA1 and HBx together into nude mice, and monitored the tumour growth rate and tumour size. Compared with negative control group, knockdown of UCA1 or HBx significantly suppressed tumour growth. It seemed that HBx knockdown displayed stronger tumour-suppressive effects than that of UCA1 knockdown (Fig. 7 and Fig. S7). Obviously, simultaneous knockdown of UCA1 and HBx further inhibited tumours formation.

## Discussion

Although it is widely accepted that HBx plays crucial role in HBV-associated HCC development, but little is known about the role of lncRNAs in the HBx-related HCC. It is still a mystery whether and how HBx promotes carcinogenesis and tumorigenesis through lncRNAs. In this study, we performed a comprehensive study to address this question via using hepatic LO2 cells and HBx-expressing hepatoma cells. We showed that UCA1 was one of the most upregulated lncRNAs by HBx. For the first time, we demonstrated that UCA1 promoted cell growth and inhibited apoptosis through the signaling of HBx-UCA1/EZH2-p27 axis.

Although majority of transcripts in human transcriptome are not translated into proteins, they play important roles in a variety of biological processes^35^,^36^. LncRNAs, a large subgroup of the non-coding transcripts, recently attract great interest. LncRNA UCA1 was first characterized as sensitive and specific marker for human bladder carcinoma and UCA1 was rarely detected in 16 normal adult tissues but highly in bladder carcinoma tissues^37^. And it was shown that UCA1 expression was elevated in a number of human tumours such as breast cancer^38^, colorectal cancer^39^, oesophageal squamous cell carcinoma^40^ and ovarian cancer^41^. Moreover, UCA1 could enhance the chemosensitivity of bladder cancer cells through caspase 3-dependent apoptosis^42^ or Wnt6-dependent Wnt signaling^43^. In addition, UCA1 promoted glycolysis by upregulating hexokinase 2 in bladder cancer cells^44^. However, UCA1 was upregulated in HCC tissues and promoted HCC metastasis through FGFR-ERK signaling pathway in recent study^45^. The mechanism of UCA1 upregulation and its other biological functions are largely unknown in HCC development. We report here, for the first time, that HBx upregulated UCA1 level in HCC cells (Figs 1 and 2), and UCA1 expression level was positively associated with the mRNA level of HBx in HCC specimens (Fig. 2). UCA1 promoted cell proliferation, decreased cell apoptosis and accelerated cell cycle progression. Cell cycle dysregulation is usually mediated by alterations in cyclin-dependent kinases (CDKs) activity. We postulated that UCA1 might be capable of regulating CDKs. Strikingly, we observed that only CDK2 specifically decreased or elevated upon UCA1 ectopic expression or knockdown in HCC cells; while other CDKs (CDK4, CDK6) were not affected by UCA1 (Fig. 5). CDK2 plays a crucial role in cell growth due to involve in both cell cycle progression and apoptotic response^30^, and its activity is regulated by CDK inhibitors p21 and p27^31^. Although CDK2 mutations are rare in human cancers; p21 and p27, the key factors in cell cycle G1/S checkpoint, are often silenced^46^. We hypothesized that UCA1 might enhance CDK2 expression or activity by repressing the expression of p21 or p27. Our results showed that UCA1 selectively suppressed p27 expression in both mRNA and protein levels without affecting p21 expression in HCC cells (Fig. 5). More importantly, we observed an inverse correlation between the p27 mRNA expression and UCA1 in HCC tissues (Fig. 5h), and silence of UCA1 in HBx-expressing hepatoma cells leading to suppression of tumour growth in nude mice (Fig. 7). These demonstrated the function of UCA1 in promoting cancer development.

To identify the underlying mechanism of UCA1 suppressing p27 expression, we conducted comprehensive studies. Firstly, we examined the distribution of UCA1 in HCC cells. We showed that UCA1 localized both in the cytosol and nucleus (Fig. 6), implying its broad biological functions. As one of the core proteins of polycomb repressive complex 2 (PRC2) and an epigenetic gene silencer, EZH2 mediates the histone methyltransferase activity and contributes to silence of tumour suppressor genes by trimethylation of lysine 27 on histone H3 across its promoter^32^. Cells treated with EZH2 inhibitors or EZH2 siRNA displayed a considerable increase of p27 expression^33^,^34^,^47^. Therefore, we hypothesized that UCA1 might suppress p27 through interacting with EZH2. Subsequently, there was no significant difference in the expression of p27 between UCA1^+^EZH2^−^ group (UCA1 was overexpressed in the absence of EZH2 expression) and UCA1^−^ group (knockdown of UCA1). Although the expression of UCA1 was upregulated, p27 expression did not reduce due to the absence of EZH2. Futhermore, we demonstrated that UCA1 repressed p27 expression via recruiting EZH2 and elevating its 3MeK27H3 level across the promoter via RIP and CHIP assays.

As an oncoprotein, HBx plays a crucial role in the development of HCC, its underlying mechanism remains to be imperfectly elucidated. Increasing evidence have revealed the important regulation role of lncRNAs in tumorigenesis, while only a few functional lncRNAs were identified in HCC. Whether and how HBx participates in tumorigenesis through lncRNAs need to be elucidated. Although we provided evidence to show that HBx upregulated UCA1 and UCA1 functioned as an oncogene in this study, we noted that UCA1 only elevated in a portion of hepatoma cell lines. On the other hand, some non-HBx-expressing hepatoma cells (e.g. Huh-7, HepG2) also expressed UCA1 (Fig. 2b). It suggested that other unknown factors could be involved in regulating UCA1 expression. In other words, there are additional mechanisms responsible for the UCA1-induced hepatocarcinogenesis which are independent on HBx. However, the results are in accordance with the extremely complicated mechanisms of hepatocarcinogenesis. Furthermore, we also noticed that HBx exhibited much stronger suppressive effects on p27 expression than UCA1 (Fig. 5), indicating that HBx-mediated hepatocarcinogenesis partly undergoes UCA1 upregulation.

In summary, we discovered that UCA1, upregulated by HBx, displayed a crucial role in G1/S transition in both hepatic and hepatoma cells. More importantly, a positive correlation between the expression of UCA1 and HBx and a negative correlation between UCA1 and p27 were observed in HCC specimens, suggesting the significance of UCA1 in HBx-mediated hepatocarinogenesis. Results from animal studies showed that UCA1 had the ability of tumorigenicity using Hep3B cells injection although we failed in finding any correlation between UCA1 expression and tumour size or survival in specimens (Fig. 7). Probably, this is due to the fact that development and progression of HCC is a complicated process driven by multiple genes and follows multiple steps as other malignance. The tumorigenicity experiment in immunodeficient nude mice regardless of the effects by other factors and is different from the complex human body. Applying loss-of-function and gain-of-function approaches, we demonstrated that UCA1 repressed p27 expression at least partly through associating with chromatin-modifying complexes PRC2 component EZH2 in HCC cells. For the first time, we demonstrated an important mechanism of hepatocarcinogenesis through the signaling of HBx-UCA1/EZH2-p27Kip1 axis, and a potential target for HCC patients.

## Methods

### Ethics statement

Nude mice were purchased from Yangzhou university medical centre (Licence No. SCXK 2012-0004, Yangzhou, China). All animal experiments were conducted in accordance with the institutional standard guidelines of Medical School of Southeast University and all experimental protocols were approved by the Use Committee for Animal Care of Jiangsu Province.

### HCC Patient Specimens

A total of 60 paired tumorous and adjacent non-tumorous liver tissues were obtained immediately after surgical resection and stored at −80 °C for further analysis from the First Affiliated Hospital of Nanjing Medical University. This study was performed with the approval of the Medical Ethical Committee of Medical School of Southeast University and every patient had written informed consent. The methods were carried out in accordance with the approved guidelines. Clinical characteristics of all patients are listed in Table 1.

### Microarray LncRNA Expression Analysis

LO2 cells stably transfected with HBx-expressing plasmids or control vectors in three biological replicates. Two HBx transfectants (LO2-HBx no. 1 and LO2-HBx no. 8), LO2-HBx stable cell mix and three LO2-Vector control cell lines were generated and used in this study. LncRNA expression profiles were analysed by the Agilent Array platform. Briefly, the total RNA extracted from cells was amplified and transcribed into fluorescent complementary DNA (cDNA), then hybridized with the 8 × 60 K LncRNA Expression Microarray (Arraystar human LncRNA Microarray V2.0). The arrays were scanned by the Agilent Scanner G2505B, and the acquired array images were analysed by Agilent Feature Extraction software (version 10.7.3.1). Data were then normalized and subsequent processed using the GeneSpring GX v11.5 software package. At last, lncRNAs with fold change ≥2.0 and *p* value <0.05 were regarded as differential expressed lncRNAs.

### Cell culture

Immortalized normal human liver cell line LO2 were obtained from the Institute of Virology, Chinese Academy of Medical Sciences (Beijing, China). LO2-Vec (LO2 stably transfected with the empty pcDNA3.1 plasmid) and LO2-HBx (LO2 stably transfected with the pcDNA4/TO-HBx plasmid) were gifts of Professor Guan Xinyuan in the University of Hong Kong. The cells were grown in RPMI Medium 1640 (Gibco, CA, USA) supplemented with 10% foetal bovine serum, 100 U/ml penicillin and 100 mg/ml streptomycin in a humidified incubator of 5% CO_2_ and 95% air at 37 °C.

### Plasmid construction

The cDNA of lncRNA-UCA1 was isolated by reverse-transcription PCR (RT-PCR) and then cloned into the Hind III/EcoR I sites of pcDNA3.1. The primer sequences are shown in Supplementary Table 1. DNA segments of UCA1-shRNA were synthesized by GENEWIZ (Suzhou, China), and ligated into the Bgl II/Hind III sites of pSUPER-EGFP vector after annealing.

### Cell Transfection

Transfections were performed using Lipofectamine 2000 according to the manufacturer’s instructions (Invitrogen, CA, USA). LO2 and Hep3B cells were transfected with 0, 1 or 2 μg of the pcDNA4/TO-HBx plasmid in 6-well plates. LO2-HBx #1 and #8 were two isolates with stable expression of HBx. The stable cell lines were also obtained by transfection of pSUPER-EGFP-UCA1 and the control vector pSUPER-EGFP cells (LO2-HBx shUCA1 and LO2-HBx shNC) and selected with G418. X-tremeGENE siRNA Transfection Reagent (Roche) was used for siRNA transfection according to the manufacturer’s protocol. LO2-HBx and Hep3B cells were transfected with HBx-specific siRNA at final concentrations of 0, 40 or 80 nM. The siRNAs were synthesized by GenePharma (Shanghai, China). The sequences of siRNAs are shown in Supplementary Table 1.

### Cell proliferation assays

The cells were plated in 96-well plates, and measured using Cell Counting Kit-8 (Dojindo Laboratories, Kumamoto, Japan). Cell proliferation was determined every 24 h for 4 days following the manufacturer’s protocol. The optical density was measured with a microplate reader (Bio-Rad, Hercules, CA, USA). For the colony formation assay, 1.5 × 10^3 ^cells were seeded in a six-well plate. After 14 days, colonies were fixed and stained with 0.05% crystal violate (Invitrogen, Carlsbad, CA, USA) before counting. Triplicate independent experiments were performed.

### Reverse transcription PCR (RT-PCR) and Real-time PCR

Total RNA was isolated using TRIzol reagent (Invitrogen, USA). The first-strand cDNA was synthesized from 1μg of total RNA using Oligo(dT) and random 6 mers oligos for priming (Takara, Dalian, China). The real-time PCR was carried out using the SYBR Premix Ex Taq (Takara, Dalian, China) according to the manufacturer’s protocol with StepOne Plus system (Applied Biosystems, Foster City, CA). The real-time PCR reactions were performed in triplicate and β-actin was used as the internal control. The relative level of target RNAs was evaluated by the comparative CT method 2^−ΔΔCT^ method. The primer sequences of each gene are shown in Supplementary Table 1.

### Western blot analysis

Cells were harvested and lysed in 0.2 ml of ice-cold lysis buffer (10 mmol/L Tris-Hcl (pH 8.0), 150 mmol/L NaCl, 1 mmol/L phenylmethylsulfonyl fluoride, and 1% Triton X-100). Total proteins were quantified using a BCA Protein Assay Kit (Beyotime, Jiangsu, China) and an equal amount of cellular proteins was separated by sodium dodecyl sulfate-polyacrylamide gel electrophoresis (SDS–PAGE) on 10% or 12% resolving gels. Proteins were transferred onto PVDF membranes and incubated for 1 hour at room temperature in block buffer (5% nonfat milk in Tris-Base Tween-20 (TBST)), then incubated overnight at 4 °C with specific primary antibodies against His tag (Fusion expression with HBx), p21, p27, CDK2, caspase-3, caspase-8, caspase-9 (from Cell Signaling Technology, USA), EZH2 (from Abcam, USA), or β-actin (from sigma, USA). The signals were detected with horseradish peroxidase-conjugated secondary antibodies (1:8000, Sigma, USA). The β-actin protein was used as an internal control. Each experiment was repeated at least 3 times.

### Flow cytometry analysis

Cells were synchronized by starvation with 1% FBS for 3 days, and harvested at 24 or 48 hours after transfection. The cells were then fixed with 70% alcohol for 1 day at −20 °C, and stained with 50 ug/ml of propidium iodide (PI) for 30 minutes before applied for flow cytometry assay. The apoptotic cells were measured by using an Annexin V-FITC Apoptosis Detection kit (BD Biosciences, San Diego, CA) according to the manufacturer’s instructions. Briefly, cells were harvested and washed twice with cold phosphate-buffered saline, incubated in the dark at room temperature (25 °C) with annexin V-FITC and propidium iodide for 10 min, and then analysed by flow cytometry assay. The annexin positive cells were considered as apoptotic cells.

### Subcellular fractionation location

RNA of nuclear and cytosolic fractions were separated using the PARIS Kit (Life Technologies, Carlsbad, CA, USA) according to the manufacturer’s instructions. Briefly, separated nuclear and cytoplasmic lysate were prepared firstly. 10^7^ fresh cultured cells were collected and placed on ice, resuspend cells in 500 μL ice-cold cell fractionation buffer and incubated on ice 10 min. Then the products were centrifuged and carefully aspirated the cytoplasmic fraction away from the nuclear pellet, and then nuclear pellet were lysed with 500 μL cell disruption buffer. Next, follow-up procedures were isolation of the nuclear and cytoplasmic RNA, and RNA were eluted with 40 μL elution solution, respectively. Equal volume of cDNA was used for subsequent real-time PCR reactions (SYBR Premix Ex Taq, TaKaRa) after the reverse transcription reaction.

### Chromatin immunoprecipitation (ChIP)

The ChIP experiments were performed using an EZ ChIP™ Chromatin Immunoprecipitation Kit for cell line samples (Millipore, USA) according to the manufacturer’s instructions. Briefly, the crosslinked chromatin DNA were sonicated into 200 to 1000 bp fragments followed by the fixed with 1% formaldehyde. Then immunoprecipitation using an anti-H3K27me3 (Millipore, USA) antibody, Histone H3 (Abcam, USA) and normal mouse IgG used as the negative control. The primers used for the amplification of p27 and Foxc1 promoter DNA fragments are listed in Supplementary Table 1.

### RNA immunoprecipitation (RIP) and RIP-PCR

RIP experiments were performed using a Magna RIP RNA-Binding Protein Immunoprecipitation Kit (Millipore, USA) according to the manufacturer’s instructions. Antibody for RIP assays of EZH2 was from Abcam (ab3748). When RIP treated with RNase, lysates were incubated with RNase for 1 hours in 37 °C. The co-precipitated RNAs were detected by conventional RT-PCR and real-time PCR. The primer sequences are listed in Supplementary Table 1.

### Xenograft HCC mice model

Hep3B cells were transfected with indicated siRNA using Lipofectamine 2000 (Invitrogen). After 24h, approximately 4 × 10^6 ^cells were harvested and injected subcutaneously into 4-week-old male nude mice. To maintain the interference efficiency, equivalent 20 μg of siRNA per mice were injected twice a week. Tumour growth was measured weekly from injection. Tumour volume (V) was monitored by measuring the length (L) and width (W) with callipers and calculated with the formula 0.5 × L × W^2^. After 4 weeks, mice were sacrificed, and the tumours were excised and measured. All animal experiments were conducted in accordance with the institutional standard guidelines of Medical School of Southeast University and all experimental protocols were approved by the Use Committee for Animal Care of Jiangsu Province.

### Data Analysis

Correlations between the UCA1 levels and pathological features were analysed with the chi square (χ^2^) test using SPSS 16.0 software for Windows. Differences were analysed by Fisher’s exact test. The Student t-test was used to compare the results, and expressed as mean ± SD between any two preselected groups or one-way analysis of variance (for three or more conditions). Pearson’s correlation coefficient was calculated using Prism5 software (GraphPad). A *P*-value <0.05 was considered statistically significant.

## Additional Information

**How to cite this article**: Hu, J.-J. *et al*. HBx-upregulated lncRNA UCA1 promotes cell growth and tumorigenesis by recruiting EZH2 and repressing p27Kip1/CDK2 signaling. *Sci. Rep*. **6**, 23521; doi: 10.1038/srep23521 (2016).

## Supplementary Material

Supplementary Information

## Figures and Tables

**Table 1:** Clinical characteristics of 60 HCC patients according to lncRNA UCA1 expression.

Factors	lncRNA-UCA1#		Chi-square	*P* Value
	Low	High		
All cases	30	30		
Age			0.635	0.426
<60	17	20		
≥60	13	10		
Gender			0.162	0.688
Male	27	26		
Female	3	4		
AFP (μg/L)			0.815	0.367
<20	3	9		
≥20	7	10		
Size (cm)			0.115	0.734
<5	12	12		
≥5	18	15		
Differentiation			0.069	0.793
I/II	13	12		
III/IV	17	18		
HBsAg			2.455	0.117
Yes	26	25		
No	4	5		
HBx			4.800	0.028*
Yes	16	24		
No	14	6		

^#^The median expression level of UCA1 was used as the cutoff. The expression of lncRNA-UCA1 in 60 patients below the 50th percentile defined as low expression and at or above the 50th percentile defined as high expression. Abbreviations: AFP, alpha-fetoprotein; HBsAg, hepatitis B surface antigen.
