# Supplementary material for: HBx-upregulated lncRNA UCA1 promotes cell growth and tumorigenesis by recruiting EZH2 and repressing p27Kip1/CDK2 signaling
Source: Sci Rep. 2016 Mar 24;6:23521. doi: 10.1038/srep23521 (PMC4806364; doi:10.1038/srep23521)
Supplement: Supplementary Information [file srep23521-s1.pdf]

Supplementary Information

HBx-upregulated lncRNA UCA1 promotes cell growth and tumorigenesis by recruiting EZH2 and repressing p27Kip1/CDK2 signaling

Jiao-Jiao Hu[1], Wei Song[1], Shao-Dan Zhang[1], Xiao-Hui Shen[1], Xue-Mei Qiu[1], Hua-Zhang Wu[1], Pi-Hai Gong[1], Sen Lu[3], Zhu-Jiang Zhao[1], Ming-Liang He[2]*, Hong Fan[1]*

Table of contents

Supplementary Figure..2

Supplementary Table..8

Supplementary Figure

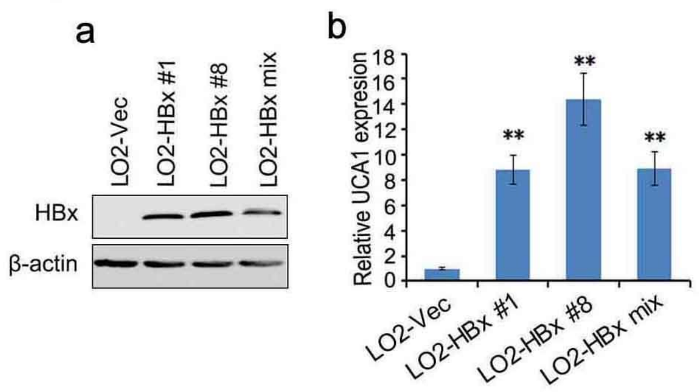

Supplementary Fig. S1, related to Fig. 2. The expression levels of HBx and UCA1 in LO2 cells. (a) The HBx protein levels detected by western blot with the anti-his tag antibody. (b) The levels of UCA1 mRNA in LO2-HBx stable cells measured by qRT-PCR.

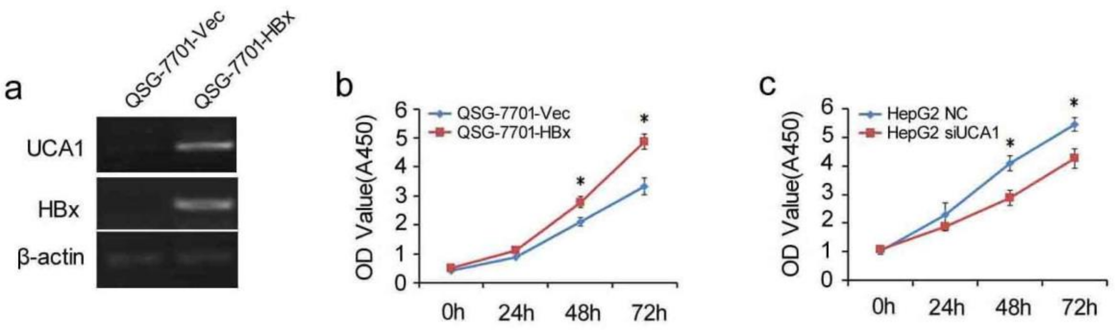

Supplementary Fig. S2, related to Fig. 3. The Role of UCA1 in cells proliferation. (a) The mRNA expression of HBx and UCA1 were detected by conventional RT-PCR. Cell proliferation was measured by Cell Counting Kit-8 assays. (b) QSG-7701 were overexpressed with HBx. (c) HepG2 cells were transfected with UCA1-specific siRNA. All data represent mean ± SD, n=3, *, P <0.05 by Student's t-test.

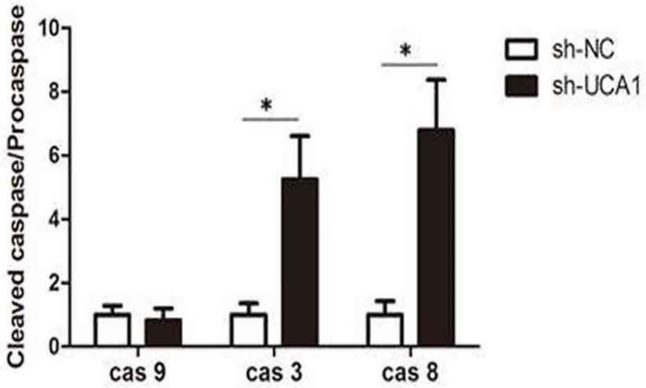

Supplementary Fig. S3, related to Fig. 4. The quantification of the western blot signals are analyzed for Figure 4c. All data represent mean ± SD, n=3, *, *P* <0.05 by Student's t-test.

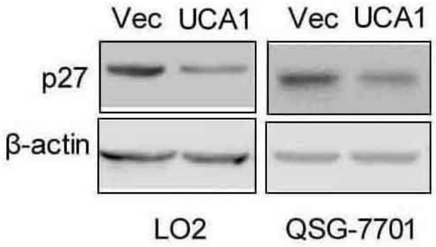

Supplementary Fig. S4, related to Fig. 5. The p27 protein levels detected by western blot with the anti-p27 antibody.

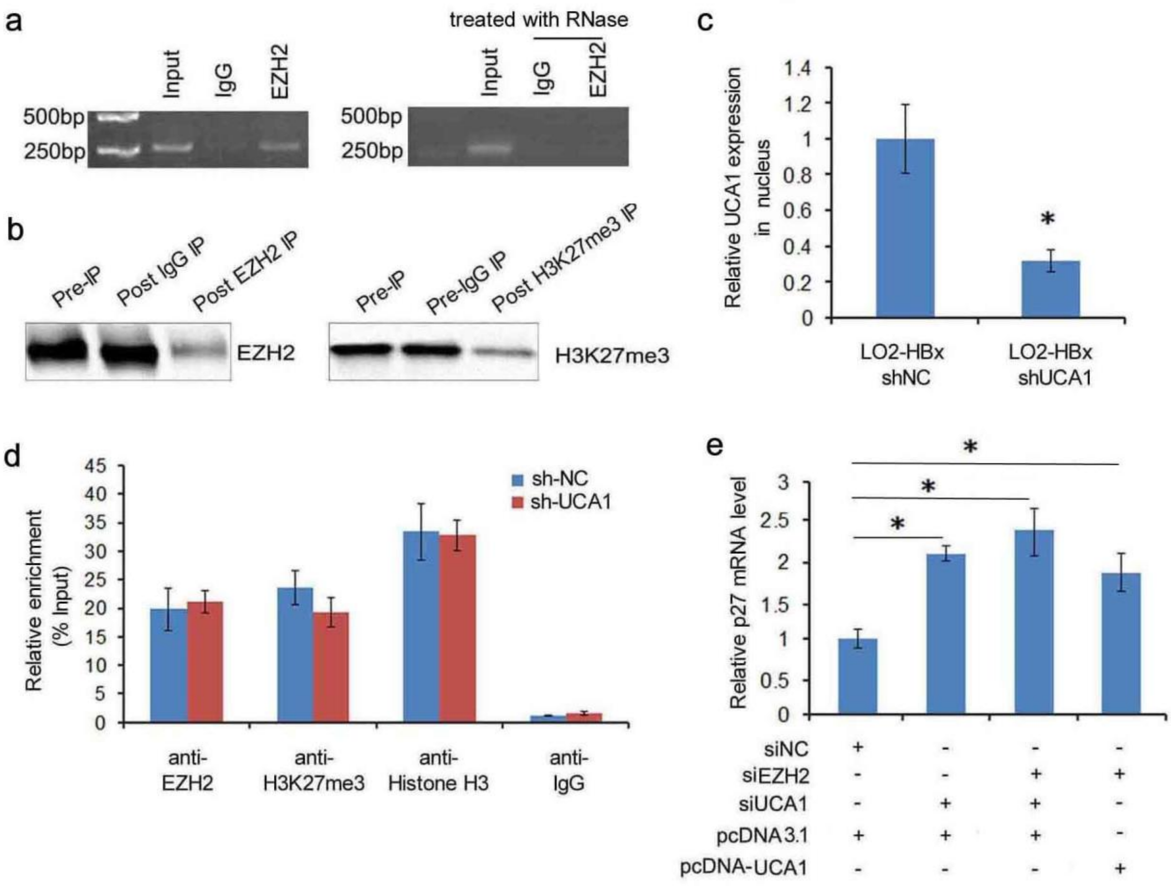

Supplementary Fig. S5, related to Fig. 6. The physical association of UCA1 and EZH2 elevating p27 3MeK27H3 level in promoters. (a) RIP assys were carried out without (left) or with (right) RNase treated. UCA1 were detected by conventional RT-PCR. (b) Before and after immunoprecipitation of CHIP, western blot experiments were carried. The results showed that EZH2 and H3K27me3 were efficiently immunoprecipitated from the cell lysates. The data are representative of two independent experiments. (c) The UCA1 expression level in cells nuleus were measured by qRT-PCR. (d) ChIP assys.Cell lysates from LO2-HBx cells without and with UCA1 knockdown were applied for immunoprecipitation using anti-EZH2, H3K27me3, Histone H3 and IgG antibodies. Foxc1 promoter was quantified by qRT-PCR. The values indicate the mean ± SD. Statistically significant differences are reported for two independent experiments. (e) The levels of p27 mRNA were detected by qRT-PCR in HepG2 cells transfected with indicated siRNA, or

UCA1-expressing plasmids, respectively. Student t-test was conducted for statistic assays. At least two independent experiments were conducted. *, $P < 0.05$.

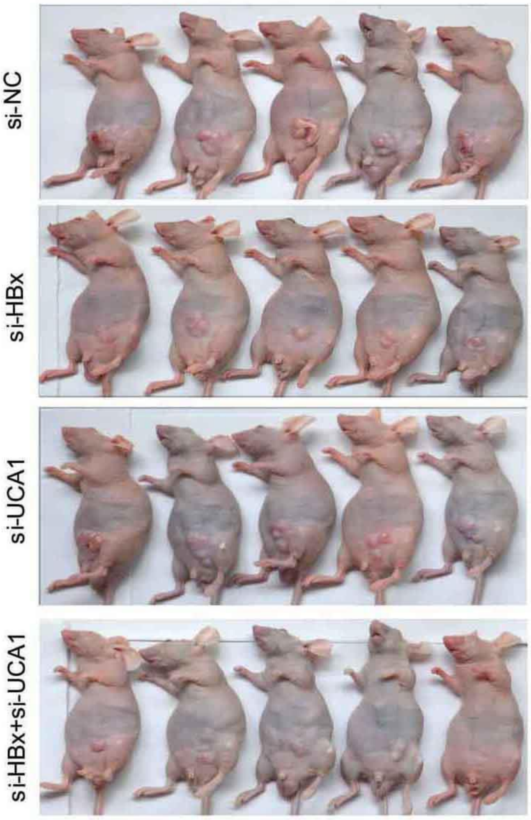

Supplementary Fig. S6,related to Fig. 7. Images of tumors in the nude mice injected with the indicated hepatoma cells after 4-week inoculation.

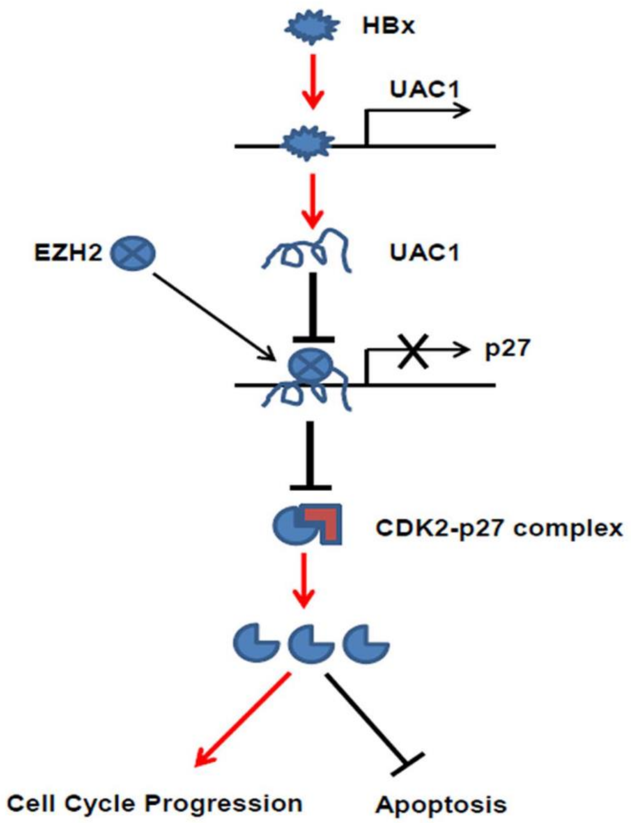

Supplementary Fig. S7. A diagram of HBx-UCA1-p27 axis signaling.

HBx activiates UCA1 expression, then UCA1 recruits EZH2 to p27 promoter and suppresses p27 expression. The reduced p27 protein level leads to high level active CDK2, that leads to G1/S transition and inhibition of apoptosis. →, activation; --|, inhibition.

Supplementary Table 1

Supplementary Table. Oligonucleotide Sequences used in this study.

Gene	Sequence (5′to 3′)	products size (bp)
ENST0000423943	F:CTCCTTTGATTTGGCTGAC	124
	R:GACCGAGACTCCATCCTG	
LOC145474	F:TATCTTTAGGCGTGGTG	184
	R:GGTATAAGGTTGGCACTA	
LOC643837	F:CTCGGCTTCTTCCTCTGTGC	138
	R:TCATCCATACACCCTTGTGACTT	
LOC100129955	F:GCCGAAGGACAAAGAGTGAA	285
	R:CGCCAAATGGGAAGCAA	
KRT42P	F: GCTCAGCAAGAAAGCATCGC	141
	R: TCCTGGTGCTCCGTGTCG	
SPRR2C	F: AGACTCCAGCAGGATCAATGTAA	178
	R: AGACTCCAGCAGGATCAATGTAA	
CEACAMP10	F: TGTCGGCATCCTGATTGG	159
	R: AGTGGGTCTTGCTCTTCGTG	
ENST00000455598	F: TGCCTCATGGAGCTGTCGTA	163
	R: TGCTATTGCTGGAGCGTAGA	
OR7E25P	F: TCGCATGTATGGAAAGTC	193
	R: TCGTTGAAGCAGGTAAGT	
HBx	F: TTCTTCGTCTGCCGTTCC	201
	R: TCGGTCGTTGACATTGCT	
HBx-97	F:GACGTCCTTTGTCTACGTCC	97

	R:GAACGGCAGATGAAGAAGGG	
UCA1	F:CTCTCCATTGGGTTCACCATTC	254
	R:GCGGCAGGTCTTAAGAGATGAG	
P27	F:CCGGCTAACTCTGAGGACAC	120
	R:AGAAGAATCGTCGGTTGCAG	
P21	F:TGATTAGCAGCGGAACAAG	218
	R:AACAGTCCAGGCCAGTATG	
EZH2	F:GAAGCAGGGACTGAAACGG	170
	R:ATTGAGGCTTCAGCACCACT	
ACTB	F:GTCATTCCAAATATGAGATGCGT	125
	R:GCTATCACCTCCCCTGTGTG	
U6	F: CGCTTCGGCAGCACATATACTA	90
	R: CGCTTCACGAATTTGCGTGTCA	
HOTAIR	F: GGTAGAAAAAGCAACCACGAAGC	170
	R: ACATAAACCTCTGTCTGTGAGTGCC	
GAPDH	GCACCGTCAAGGCTGAGAAC	138
	TGGTGAAGACGCCAGTGGA	
UCA1 clone	F:CCCAAGCTTTGACATTCTTCTGGAC	1423
	R: GGAATTCAATCAGGCATATTAGCTT	
CHIP p27 Pa	F: TCTGGGTTAAGGCTGAGCGA	206
	R: GAGGACCGCGAAGGTCG	
CHIP p27 Pb	F: GCTCCTAGCGAGCTGCC	204
	R: GGCTGGCCTCGGAGAAATTA	
CHIP p27 Pc	F:TTTTAATTTCTCCGAGGCCAGC	207

	R:GAGATCCATTGGTTGCGGCG	
CHIP p27 Pd	F:TTCTTCGTCAGCCTCCCTTC	170
	R: AGCCGCTCTCCAAACCTTG	
CHIP p27 Pe	F: CTGGTCCCCTCTCCTCTCC	177
	R: CGAACCCCTCTCGGAAGC	
CHIP p27 Pf	F: GCTTCCGAGAGGGGTTCG	194
	R: GGTTCCTGCAGGCCGAG	
CHIP p27 Pg	F: GATGGACGCCAGGCAGG	204
	R: CTGCCCTTCTCCACCTCTTG	
CHIP p27 Ph	F: CCCCTAGAGGGCAAGTACGA	167
	R: TTAGCCGGAGCCCCAATTAAA	
CHIP Foxc1	F:ACCCCTTGCCTTCATTTGG	218
	R:AGCCCCTTCCCTCCCTTCT	
**siRNA sequence**		
HBx-siRNA	Sense: CUUGGACUCUCAGCAAUGUTT	
	Antisense:ACAUUGCUGAGAGUCCAAGTT	
UCA1-siRNA1	Sense: GUUAAUCCAGGAGACAAAGATT	
	Antisense:UCUUUGUCUCCUGGAUUAACTT	
UCA1-siRNA2	Sense: GGACAACAGUACACGCAUAdTdT	
	Antisense:TATGCGTGTACTGTTGTCCdTdT	
EZH2-siRNA	Sense: GAGGUUCAGACGAGCUGAUUU	
	Antisense:AAAUCAGCUCGUCUGAACCUC	
